# Acute and continuous exposure of airborne fine particulate matter (PM_2.5_): diverse outer blood–retinal barrier damages and disease susceptibilities

**DOI:** 10.1186/s12989-023-00558-2

**Published:** 2023-12-18

**Authors:** Yuzhou Gu, Feiyin Sheng, Mengqin Gao, Li Zhang, Shengjie Hao, Shuying Chen, Rongrong Chen, Yili Xu, Di Wu, Yu Han, Lu Chen, Ye Liu, Bing Lu, Wei Zhao, Xiaoming Lou, Zhijian Chen, Peng Li, Xiaofeng Wang, Ke Yao, Qiuli Fu

**Affiliations:** 1 Eye Center of the 2nd Affiliated Hospital, School of Medicine, Medical College of Zhejiang University, Zhejiang University, Zhejiang Provincial Key Lab of Ophthalmology, Hangzhou, 310009 Zhejiang Province China; 2 State Key Lab of Modern Optical Instrumentation, College of Optical Science and Engineering, Zhejiang University, Hangzhou, 310027 Zhejiang Province China; 3 Department of Environmental and Occupational Health, Zhejiang Provincial Center for Disease Control and Prevention, Hangzhou, 310051 Zhejiang Province China

**Keywords:** PM_2.5_, Retinal pigment epithelium, Choriocapillaris, Inflammation, Outer blood–retinal barrier

## Abstract

**Background:**

The association between air pollution and retinal diseases such as age-related macular degeneration (AMD) has been demonstrated, but the pathogenic correlation is unknown. Damage to the outer blood–retinal barrier (oBRB), which consists of the retinal pigment epithelium (RPE) and choriocapillaris, is crucial in the development of fundus diseases.

**Objectives:**

To describe the effects of airborne fine particulate matter (PM_2.5_) on the oBRB and disease susceptibilities.

**Methods:**

A PM_2.5_-exposed mice model was established through the administration of eye drops containing PM_2.5_. Optical coherence tomography angiography, transmission electron microscope, RPE immunofluorescence staining and Western blotting were applied to study the oBRB changes. A co-culture model of ARPE-19 cells with stretching vascular endothelial cells was established to identify the role of choroidal vasodilatation in PM_2.5_-associated RPE damage.

**Results:**

Acute exposure to PM_2.5_ resulted in choroidal vasodilatation, RPE tight junctions impairment, and ultimately an increased risk of retinal edema in mice. These manifestations are very similar to the pachychoroid disease represented by central serous chorioretinopathy (CSC). After continuous PM_2.5_ exposure, the damage to the RPE was gradually repaired, but AMD-related early retinal degenerative changes appeared under continuous choroidal inflammation.

**Conclusion:**

This study reveals oBRB pathological changes under different exposure durations, providing a valuable reference for the prevention of PM_2.5_-related fundus diseases and public health policy formulation.

**Graphical abstract:**

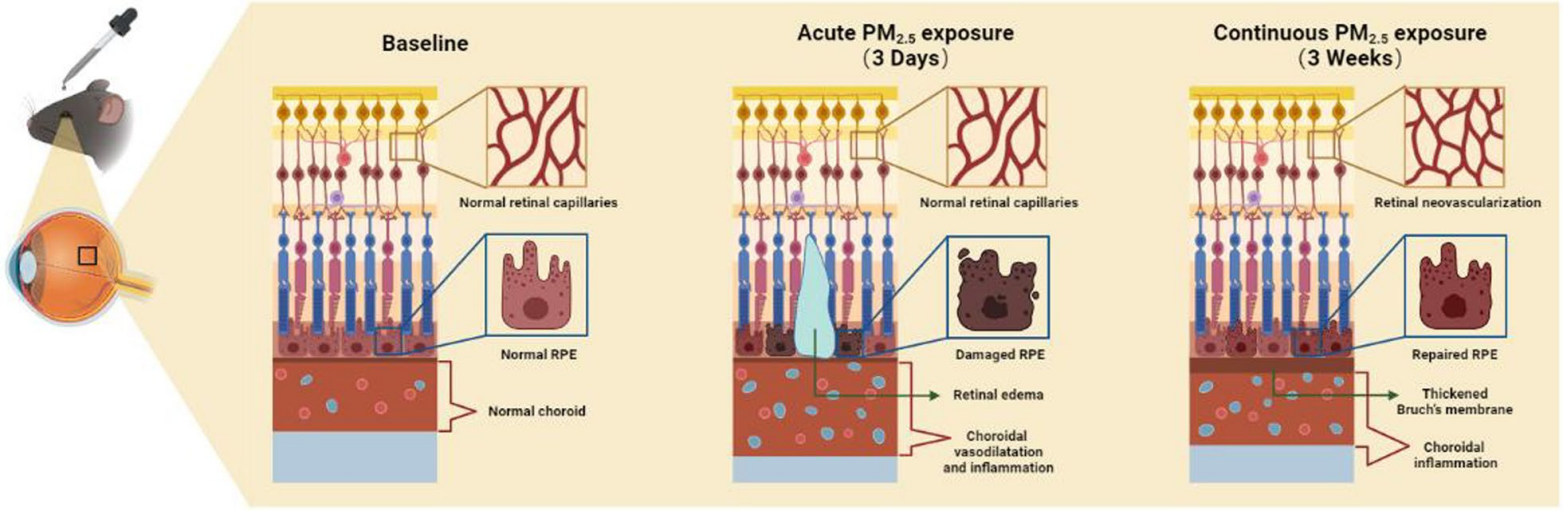

**Supplementary Information:**

The online version contains supplementary material available at 10.1186/s12989-023-00558-2.

## Background

Air pollution is an environmental problem that also affects human health and has been proven to have a clear correlation with many diseases [1–3]. In the field of ophthalmology, there is sufficient evidence to support the association of ocular surface diseases, such as dry eye and conjunctivitis, with air pollution [4–7], while there are relatively few studies on retinal diseases due to the indirect contact of the retina with air. The retina is crucial to vision formation, in which the neuroepithelial layer is photoreceptive and basically transparent except where blood vessels are distributed. Due to the highly open choriocapillaris with high blood flow, and the high energy metabolism rate of the retina, retinal tissues are susceptible to damage from adverse external stimuli, including xenobiotics. Different types of retinopathy such as retinal vascular disease, macular degeneration, retinal detachment, and retinal dystrophy will occur [8]. Age-related macular degeneration (AMD) is one of the most common retinal diseases and is the main cause of irreversible vision loss in adults in developed countries [9]. At present, a number of cohort studies from different countries have found that air pollution is associated with changes in the thickness of various retinal layers, and that long-term exposure to fine particulate matter with a diameter of < 2.5 μm (PM_2.5_) is related to an elevated risk of AMD [10–13]. However, so far, there have been no studies conducted on animal models to explore the AMD-related pathologic phenotypes induced by air pollution, and no cellular models close to the environment in vivo have been established to investigate the pathogenesis of PM_2.5_-related AMD.

**Fig. 1:**
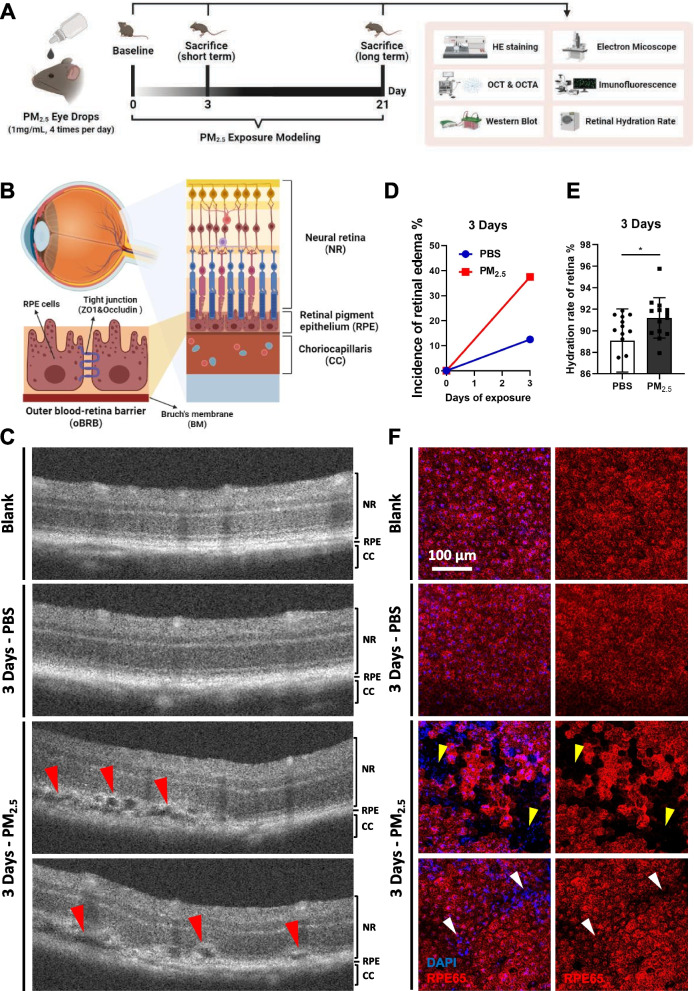
Acute PM_2.5_ exposure induces retinal edema by damaging RPE in mice. **A** Schematic diagram of PM_2.5_-exposed C57BL/6J mouse model establishment. OCT, optical coherence tomography; OCTA, optical coherence tomography angiography. **B** Schematic diagram of the structural composition of oBRB. **C** OCT images of the retina in mice after exposure of PBS or PM_2.5_ for 3 days. Retinal edema is indicated with red arrows. PBS, phosphate buffer saline. **D** Incidence of retinal edema in mice exposed to PBS or PM_2.5_ for 3 days. **E** Hydration rate of retina in mice treated with PBS or PM_2.5_ for 3 days (n = 14). **F** Immunofluorescence analysis of RPE specific marker RPE65 on mouse RPE flat-mounts. Bridging-like loss of RPE cells (yellow arrows) and enlargement of cell gaps (white arrows) are indicated. Data in (**E**) are presented as mean ± SD. *p < 0.05

Our recent study, for the first time, explored the influence of PM2.5 exposure on the retina and found that PM_2.5_ exposure damages the inner blood–retinal barrier (iBRB) [14], which is the common pathological process of various retinal vascular diseases, such as diabetic retinopathy and retinal vein obstruction [15]. However, the pathogenesis of AMD is different, mainly due to the breakdown of the outer blood–retinal barrier (oBRB) but not the iBRB [9, 16]. As shown in Fig. 1B, the oBRB is composed of retinal pigment epithelium (RPE) monolayer cells and the adjacent Bruch’s membrane, located between the neural retina and the choriocapillaris [17]. The tight junctions (TJ) between RPE monolayer cells and the complete Bruch’s membrane structure play a critical role in strictly controlling the permeability of the oBRB, selectively preventing the lipids, lipoproteins, and harmful substances in the choriocapillaris from infiltrating and depositing in the retinal tissue [18, 19]. In exudative AMD, RPE cell dysfunction, retinal or choroidal neovascularization, and diffused thickening of the Bruch’s membrane are characteristic pathological changes [16].

Central serous chorioretinopathy (CSC), a common type of retinopathy, also threatens vision [20]. Its pathogenesis is similar to that of AMD, in that there is destruction of the oBRB caused by RPE damage. This is mostly due to the abnormal expansion of choriocapillaris and choroidal thickening, which leads to blood flow overload and produces a large amount of exudative fluid breaking through the RPE layer, resulting in subretinal effusion [21–23]. But unlike the chronic course of AMD, the progression of CSC tends to be acute, with sudden vision loss and distortion. The course of CSC is often self-limited, with a self-healing rate of 60%, but there is still a high risk of recurrence [24]. At present, smoking is known to be a definite predisposing factor for CSC [24, 25]. Since PM_2.5_ is also one of the main components of cigarette smoke, there is speculation that acute air pollution may also increase the risk of CSC. However, there has never been a study analyzing the association between exposure and morbidity.

The present research aims to explore the pathological changes and interaction of the RPE and choroid under acute and continuous PM_2.5_ exposure, as well as to offer solid evidence for the correlation between air pollution and oBRB injury-related diseases such as AMD and CSC on phenotypic level, and provides a new perspective on the prevention of air-pollution-related fundus diseases.

## Results

### Acute PM_2.5_ exposure impairs the barrier function of the RPE in mice

In our previous study, we found that acute exposure to PM_2.5_ caused significant iBRB damage. The peak of retinal vascular leakage was observed after 3 days of PM_2.5_ exposure, accompanied by retinal vasodilation and inflammatory responses [14]. In further exploring what kinds of changes occurred in the oBRB under acute exposure, the details of the animal model establishment remained consistent with our previous study: mice treated with 1 mg/mL of PM_2.5_ suspension eye drops for 3 days. First, fundus cross-sectional images obtained by optical coherence tomography (OCT) (Fig. 1C) showed that 37.5% of the eyes exposed to PM_2.5_ appeared visible retinal edema above the RPE layer, much higher than the 12.5% in the PBS group (Fig. 1D). Determination of the retinal hydration rate also revealed that the amount of water in retinas was significantly higher in the PM_2.5_-exposed group than in the control group (Fig. 1E), providing strong evidence that acute exposure to PM_2.5_ increases the risk of retinal edema. To investigate the reason for the edema above the RPE layer, immunofluorescence of the RPE cell-specific marker RPE-65 was performed on RPE flat-mounts of mice (Fig. 1F). The results suggested that acute exposure to PM_2.5_ disrupted the integrity of the RPE monolayer cells in some areas, where bridging-like loss of RPE cells and enlargement of cell gaps could be observed. These experiments revealed that the dysfunction of the oBRB caused by disruption of the structural integrity of the RPE layer is an essential cause of retinal edema triggered by PM_2.5_ (Fig. 5F).

**Fig. 2:**
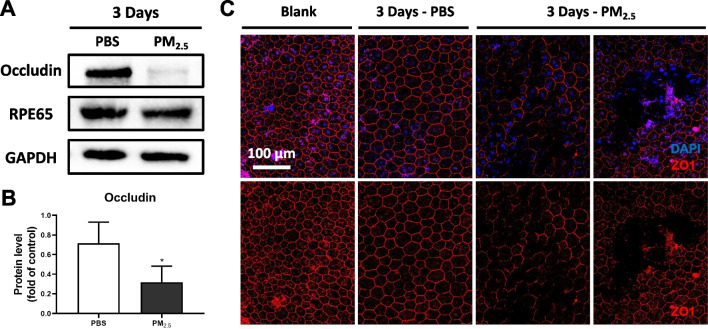
Acute PM_2.5_ exposure impairs TJs of RPE in mice. **A** Expression of RPE65 and TJ protein Occludin in mouse RPE/choroidal tissues suggested by immunoblotting. GAPDH served as the internal control. PBS, phosphate buffer saline; TJ, tight junction; GAPDH, glyceraldehyde-3-phosphate dehydrogenase. **B** Quantification of shown blot, normalized by GAPDH (n = 3). **C** Immunofluorescence staining of TJ marker ZO1 on RPE flat-mounts in mice treated with PBS or PM_2.5_ for 3 days. Data in (**B**) are presented as mean ± SD. *p < 0.05

The barrier function of the RPE is largely realized by the TJs between RPE cells. As revealed by Western blotting of Occludin, one of the TJ proteins, in the RPE layer, the content of Occludin in mouse RPE/choroidal tissues was significantly higher in the PM_2.5_-exposed group (Fig. 2A, B). Immunofluorescent staining of another kind of TJ protein, namely ZO1, in mouse RPE flat-mounts also indicated the absence of RPE intercellular TJ proteins in some areas (Fig. 2C). These findings suggest that the damage of RPE TJs under acute PM_2.5_ exposure is also an important cause of impaired barrier function of the oBRB.

### Acute PM_2.5_ exposure induces choroidal vasodilation and inflammation in mice

**Fig. 3:**
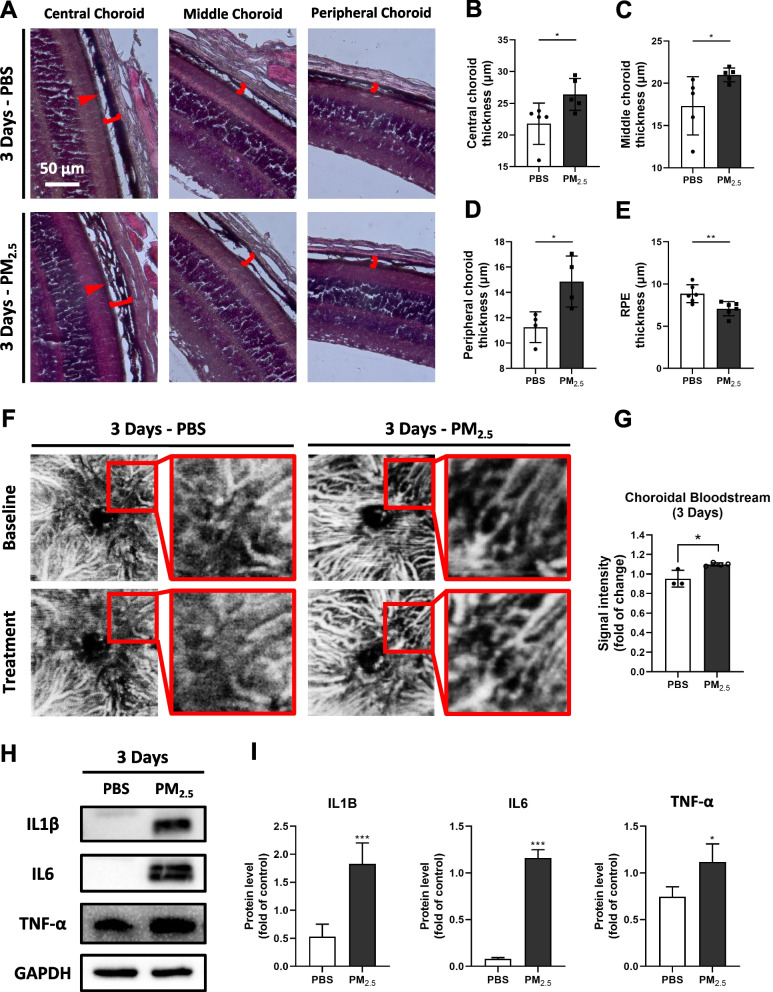
Acute PM_2.5_ exposure induces choroidal vasodilation and inflammation in mice. **A** HE staining for the morphology of the choroid (red brackets) and RPE (red arrows) in mice exposed to PBS or PM_2.5_ for 3 days. PBS, phosphate buffer saline. **B–D** Measurements of mice central (n = 5), middle (n = 5), and peripheral choroid thickness (n = 4) in HE staining images. **E** Measurements of mice RPE thickness (n = 5) in HE staining images. **F** OCTA images of the choroid blood vessels in mice before and after exposure of PBS or PM_2.5_ for 3 days. **G** The ratio of choroid blood flow signal intensity in mice before and after 3 days exposure to PBS (n = 3) or PM_2.5_ (n = 4). **H** Expression of inflammation-related proteins in mouse RPE/choroidal tissues suggested by immunoblotting. GAPDH served as the internal control. IL1B, interleukin 1 beta; IL6, interleukin 6; TNF-α, tumor necrosis factor; GAPDH, glyceraldehyde-3-phosphate dehydrogenase. **I** Quantification of shown blots, normalized by GAPDH (n = 3). Data in (**B**–**E**, **G**, **I**) are presented as mean ± SD. *p < 0.05, **p < 0.01, and ***p < 0.001

Considering the structural and functional connection between the choroid and the RPE [17, 26, 27], the study next turned to exploring the effects of acute PM_2.5_ exposure for 3 days on the choriocapillaris in mice. First, H&E staining of mouse eye sections showed a significant increase in choroidal thickness at the center, middle, and periphery during acute PM_2.5_ exposure (Fig. 3A–D). However, a significant decrease in the thickness of the RPE layer was observed (Fig. 3E). Subsequently, optical coherence tomography angiography (OCTA) imaging of choroidal blood flow showed that the blood vessels in some areas were significantly dilated after 3 days of PM_2.5_ exposure compared with pre-exposure (Fig. 3F), and overall choroidal blood flow was significantly increased (Fig. 3G). Finally, further Western blotting of inflammation-related proteins interleukin 1 beta (IL1B), interleukin 6 (IL6), and tumor necrosis factor (TNF-α) in the RPE/choroidal tissues of mice revealed that the inflammation in the choriocapillaris was significantly more serious in the PM_2.5_-exposed group than in the control group (Fig. 3H, I). These experiments convincingly demonstrated that choroidal vasodilatation occurred under acute exposure to PM_2.5_, accompanied by the development of inflammation (Fig. 5F).

### Continuous PM_2.5_ exposure causes AMD-related pathological retinal changes

**Fig. 4:**
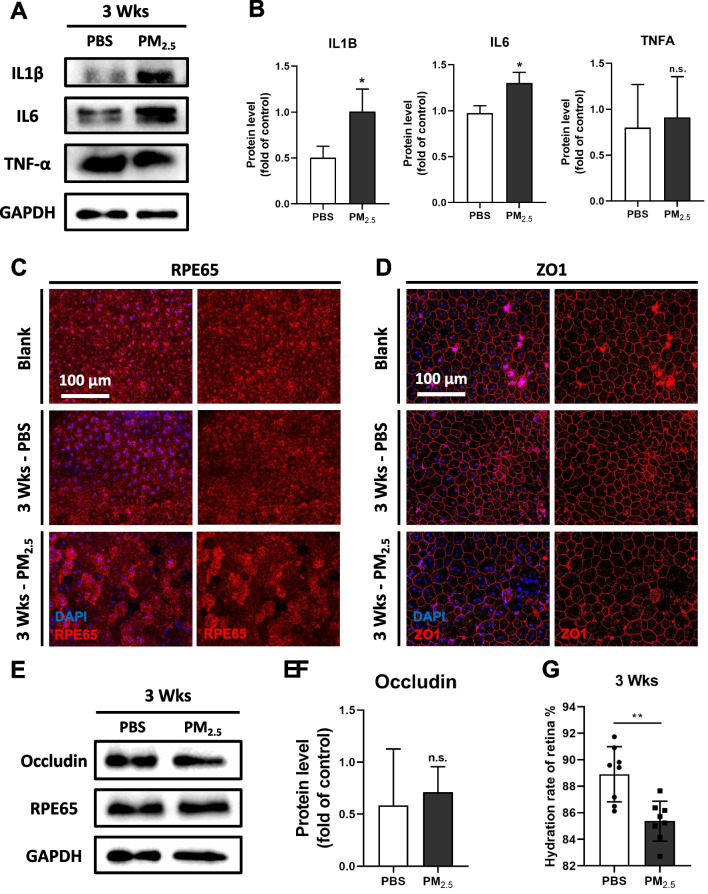
Continuous PM_2.5_ exposure prompts irregular repair of RPE barrier structure in mice. **A** Expression of inflammation-related proteins in mouse RPE/choroidal tissues under exposure of PBS or PM_2.5_ for 3 weeks suggested by immunoblotting. GAPDH served as the internal control. PBS, phosphate buffer saline; RPE, retinal pigment epithelium; IL1B, interleukin 1 beta; IL6, interleukin 6; TNF-α, tumor necrosis factor; GAPDH, glyceraldehyde-3-phosphate dehydrogenase. **B** Quantification of shown blots, normalized by GAPDH (n = 3). **C**, **D** Immunofluorescence staining of RPE65 and ZO1 on RPE flat-mounts in mice treated with PBS or PM_2.5_ for 3 weeks. **E** Expression of RPE65 and TJ protein Occludin in mouse RPE/choroidal tissues suggested by immunoblotting. GAPDH served as the internal control. **F** Quantification of shown blot, normalized by GAPDH (n = 3). **G** Hydration rate of retina in mice treated with PBS or PM_2.5_ for 3 weeks (n = 8). Data in (**B**, **F**, **G**) are presented as mean ± SD. *p < 0.05, and **p < 0.01

**Fig. 5:**
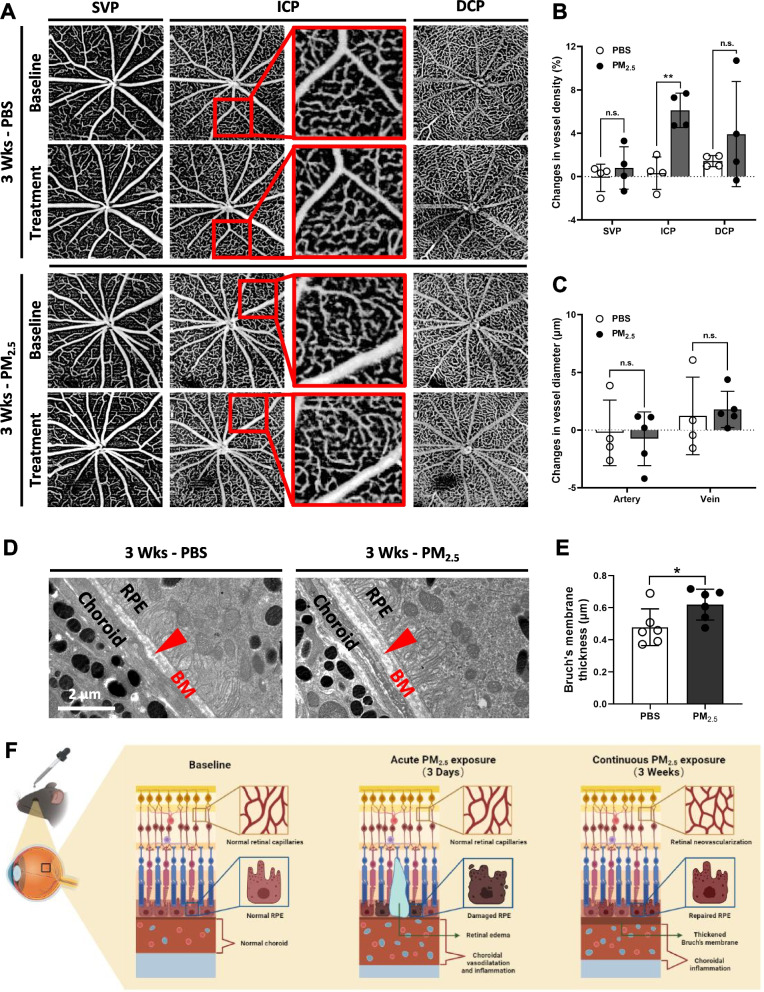
Continuous PM_2.5_ exposure causes AMD-related retinal pathological changes in mice. **A** OCTA images of the retinal blood vessels in mice before and after exposure of PBS or PM_2.5_ for 3 weeks. OCTA, optical coherence tomography angiography; AMD, age-related macular degeneration; PBS, phosphate buffer saline; SVP, superficial vascular plexus; ICP, intermediary capillary plexus; DCP, deep capillary plexus; **B** Changes in retinal vessel density compared to its baseline in mice exposed to PBS or PM_2.5_ (n = 4). **C** Changes in retinal vessel diameter compared to its baseline in mice exposed to PBS (n = 4) or PM_2.5_ (n = 5). **D** TEM images of the BM in mice after exposure of PBS or PM_2.5_ for 3 weeks. TEM, transmission electron microscope; BM, Bruch’s membrane; RPE, retinal pigment epithelium. **E** Measurements of mice BM thickness (n = 6) in TEM images. **F** Schematic diagram of fundus pathological changes in mice under acute and continuous PM_2.5_ exposure. Data in (**B**, **C**, **E**) are presented as mean ± SD. *p < 0.05, and **p < 0.01

To explore the changes in the RPE and choroid under the continuous influence of PM_2.5_, the duration of PM_2.5_ exposure on mice was extended to 3 weeks. Western blotting of inflammation-related proteins IL1B, IL6, and TNF-α in mouse RPE/choroidal tissues revealed that choroidal inflammation levels after long-term PM_2.5_ exposure remained significantly higher than those in the PBS group (Fig. 4A, B). Immunofluorescence of RPE65 on RPE flat-mounts of mice revealed that the bridging-like loss of RPE cells was largely repaired after 3 weeks of PM_2.5_ exposure (Fig. 4C), whereas immunofluorescence of ZO1 displayed irregular shape and inhomogenous size of RPE cells in the repaired area, and most of the intercellular TJs were also regained (Fig. 4D). Further Western blotting of Occludin also revealed that the TJ protein content of RPE tissues in the PM_2.5_-exposed group was no longer significantly different from that in the PBS group (Fig. 4E, F). Determination of hydration rate revealed that the amount of water in the retinas was lower in the PM_2.5_-exposed group than in the control group (Fig. 4G). Subsequently, OCTA imaging of retinal blood flow showed that retinal capillary density after 3 weeks of PM_2.5_ exposure was higher than before exposure, with statistically significant differences in the intermediary capillary plexus (ICP) (Fig. 5A, B). However, no significant changes in vessel diameter were observed (Fig. 5C), suggesting that long-term PM_2.5_ exposure promotes the process of retinal neovascularization. TEM also observed a significant thickening of the Bruch's membrane between the RPE and the choroid under continuous PM_2.5_ exposure (Fig. 5D, E). In conclusion, the above findings of retinal neovascularization and Bruch's membrane thickening are both characteristic pathological changes of AMD (Fig. 5F), indicating that there may be associations between long-term PM_2.5_ exposure and the development of AMD.

### Short-term dilated choriocapillaris induces RPE cytotoxicity and TJ disruption

**Fig. 6:**
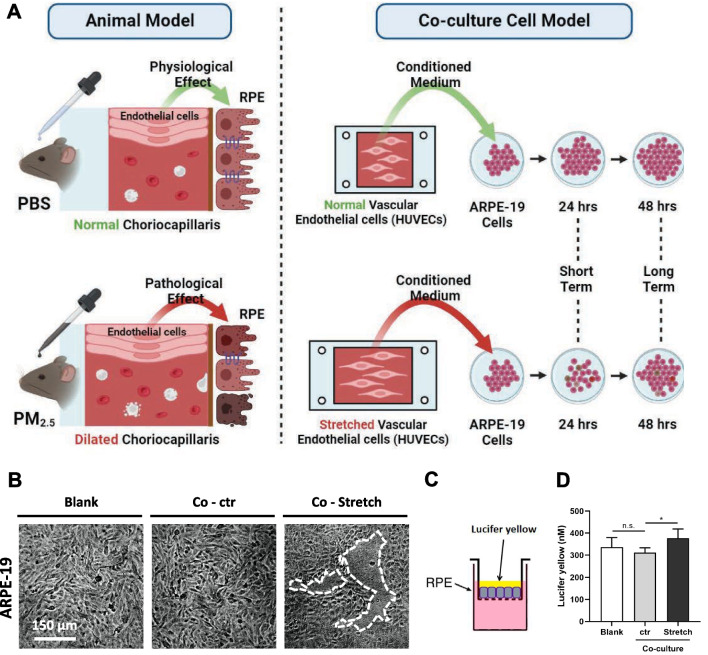
The permeability of ARPE-19 monolayer increases when affected by stretched HUVECs. **A** Schematic diagram of indirect co-culture of ARPE-19 cells and unstretched or stretched HUVECs. PBS, phosphate buffer saline; RPE, retinal pigment epithelium. **B** Images of ARPE-19 monolayers treated with conditioned medium from unstretched (Co-ctr) or stretched (Co-Stretch) HUVECs for 24 h. The blank group refers to ARPE-19 cells that have not been co-cultured. **C** Schematic diagram of ARPE-19 paracellular permeability assay conducted with Lucifer Yellow. **D** Permeability of ARPE-19 monolayer treated with conditioned medium from unstretched or stretched HUVECs for 24 h (n = 4). Data in (**D**) are presented as mean ± SD. *p < 0.05

**Fig. 7:**
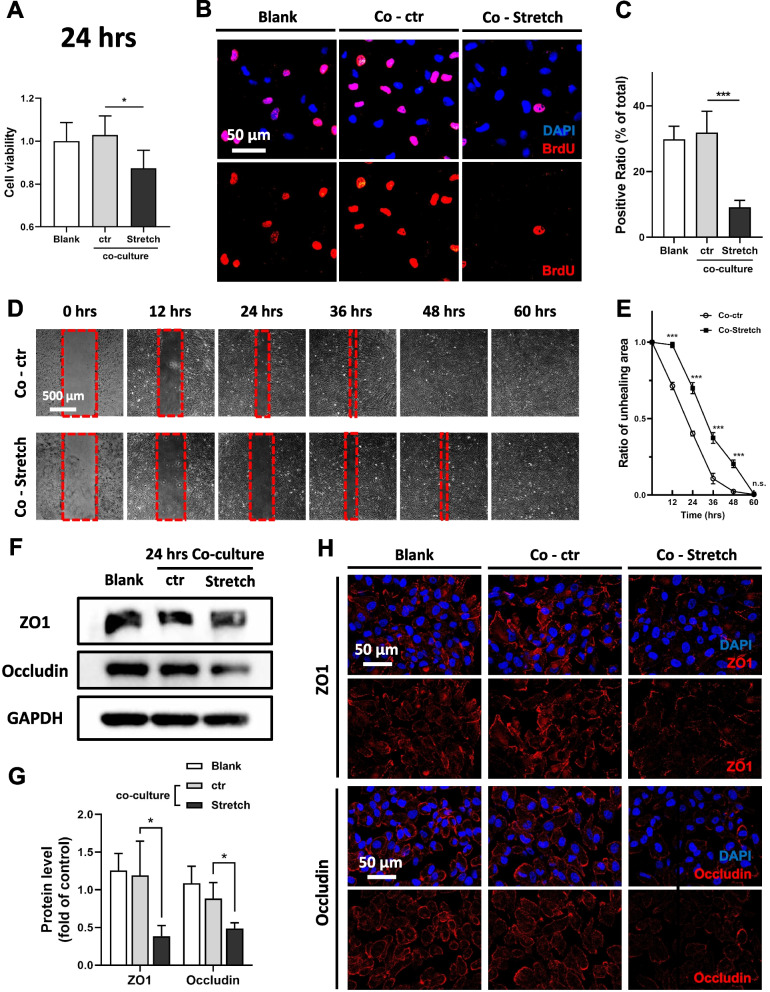
Short-term co-culture of stretched HUVECs induces ARPE-19 cytotoxicity and TJ disruption. **A** Cell viability of ARPE-19 detected by CCK-8. ARPE-19 cells are treated with conditioned medium from unstretched (Co-ctr) or stretched (Co-Stretch) HUVECs for 24 h. The blank group refers to ARPE-19 cells that have not been co-cultured (n = 5). CCK-8, Cell Counting Kit-8. **B** Images of BrdU–labeled ARPE-19 cells co-cultured for 24 h. BrdU, 5-iodine-2'-deoxyuridine. **C** Percentage of the shown BrdU+ cells (n = 5). **D** Images of scratch wound healing assay for ARPE-19 cells co-cultured for 0, 12, 24, 36, 48 or 60 h. **E** Quantification of unhealing wound area compared to the baseline (0 h, n = 4). **F** Expression of TJ proteins ZO1 and Occludin in ARPE-19 cells co-cultured for 24 h suggested by immunoblotting. GAPDH served as the internal control. TJ, tight junction; GAPDH, glyceraldehyde-3-phosphate dehydrogenase. **G** Quantification of shown blots, normalized by GAPDH (n = 3). **H** Immunofluorescence staining of TJ marker ZO1 and Occludin in ARPE-19 cells co-cultured for 24 h. Data in (**A**, **C**, **E**, **G**) are presented as mean ± SD. *p < 0.05, and ***p < 0.001

Since pathological changes in both the RPE and choroid during PM_2.5_ exposure had been identified, the next step was to explore the specific pathogenic mechanisms. The choroidal vascular endothelium, as the main site that triggers the inflammatory response, and as the inner wall of the vessels that is in direct contact with blood flow, undergoes elongation along with the dilation of the vessels [28]. Therefore, choroidal vascular endothelial cells are likely to play a key role in the series of choroidal pathophysiological changes induced by PM_2.5_ exposure. Considering that choroidal vasodilatation occurred under PM_2.5_ exposure, indirect co-culturing of ARPE-19 cells was conducted using conditioned medium from stretched human umbilical vein endothelial cells (HUVECs). In the case of short-term co-culturing for 24 h, ARPE-19 cells affected by stretched HUVECs failed to grow into intact monolayers compared to those affected by unstretched HUVECs (Fig. 6B). Paracellular permeability assay also suggested that ARPE-19 monolayers affected by stretched HUVECs resulted more Lucifer Yellow leakage (Fig. 6D), suggesting impaired RPE barrier function. Further studies revealed that ARPE-19 affected by stretched HUVECs in the short term showed less viability (Fig. 7A), and the percentage of BrdU+ cells declined, suggesting that cell proliferation was inhibited (Fig. 7B, C). In addition, scratch wound healing assays revealed that the healing speed of ARPE-19 affected by stretched HUVECs was slower than in the control group, indicating that cell migration had also been suppressed (Fig. 7D, E). As to whether TJs, which play a key role in RPE barrier function, were also adversely affected, the study further quantified and localized 2 TJ proteins, ZO1 and Occludin. Western blotting showed a significant decrease in TJ protein content in ARPE-19 affected by stretched HUVECs (Fig. 7F, G), and immunofluorescence revealed that the distribution of TJ protein was also significantly reduced in the cell membrane (Fig. 7H). Overall, the short-term influence of stretched HUVECs on ARPE-19 resulted in the inhibition of cell viability, proliferation, and migration properties, as well as the impairment of TJs, which are consistent with the findings in mice, suggesting that the short-term exposure to PM_2.5_ probably caused RPE damage by triggering choroidal vasodilation.

### Long-term dilated choriocapillaris enables adaptation and repair of RPE damage

**Fig. 8:**
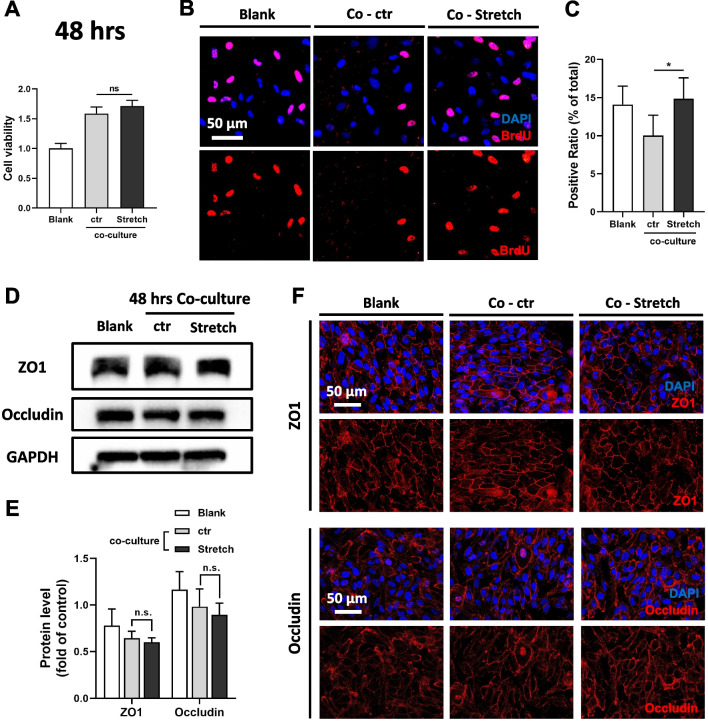
Long-term co-culture of stretched HUVECs enables repair of ARPE-19 cytotoxicity and TJ disruption. **A** Cell viability of ARPE-19 detected by CCK-8. ARPE-19 cells are treated with conditioned medium from unstretched (Co-ctr) or stretched (Co-Stretch) HUVECs for 48 h. The blank group refers to ARPE-19 cells that have not been co-cultured (n = 5). CCK-8, Cell Counting Kit-8. **B** Images of BrdU–labeled ARPE-19 cells co-cultured for 48 h. BrdU, 5-iodine-2'-deoxyuridine. **C** Percentage of the shown BrdU+ cells (n = 5). **D** Expression of TJ proteins ZO1 and Occludin in ARPE-19 cells co-cultured for 48 h suggested by immunoblotting. GAPDH served as the internal control. TJ, tight junction; GAPDH, glyceraldehyde-3-phosphate dehydrogenase. **E** Quantification of shown blots, normalized by GAPDH (n = 3). **F** Immunofluorescence staining of TJ marker ZO1 and Occludin in ARPE-19 cells co-cultured for 48 h. Data in (**A**, **C**, **E**) are presented as mean ± SD. *p < 0.05

Once the short-term influence of stretched HUVECs on ARPE-19 cells had been determined, the next step was to explore whether ARPE-19 cells acted differently when subjected to stretched HUVECs in the long term, thus simulating the changes in RPE under long-term exposure to PM_2.5_, to some extent. CCK-8 assay revealed that the viability of ARPE-19 cells under the long-term influence of stretched HUVECs (indirect co-culturing for 48 h) was not significantly different from that of ARPE-19 cells affected by unstretched HUVECs (Fig. 8A). However, the percentage of BrdU+ cells was higher in ARPE-19 affected by stretched HUVECs (Fig. 8B, C), suggesting a compensatory increase in cell proliferation level. TJ proteins (ZO1 and Occludin) were also quantified and localized, and the results showed that unlike the TJs of ARPE-19 cells that were suppressed when affected by stretched HUVECs in the short term, the expression (Fig. 8D, E) and location (Fig. 8F) of TJs under long-term co-culturing were not significantly different from those same elements in the control group. Overall, the above findings suggest that ARPE-19 cells, under the long-term influence of stretched HUVECs, somewhat adapted to the continuous stress, and the damage of TJs induced by short-term co-culturing was gradually repaired. These results also hint that long-term PM_2.5_ exposure induces different changes in the oBRB when compared to acute exposure, which is consistent with the findings in mice.

## Discussion

The present study described the effects of PM_2.5_ exposure on the RPE and choroid for the first time, and provides a detailed description of the pathological changes under two different scenarios of short-term and long-term exposure, providing evidence to support the association of air pollution with oBRB injury-related eye diseases. Acute PM_2.5_ exposure was found to cause fundus changes that are very similar to the pathogenesis and manifestations of CSC. Under continuous PM_2.5_ exposure, AMD-related pathological changes were also observed. Moreover, this study pioneered an indirect co-culture model of stretched cells, which provided insight into the changes in choroidal hemodynamics as a key mechanism of RPE damage induced by PM_2.5_ exposure.

In this study, acute PM_2.5_ exposure caused choroidal vasodilation, thickening, and inflammation in mice, along with disruption of TJs between RPE cells, leading to the appearance of retinal edema above the RPE layer (Fig. 5F). Such pathological changes are very similar to what is seen in pachychoroid disease [29, 30], which is a recent, general term for a specific group of diseases that covers diseases with the common features of choroidal hypertrophy, choroidal vasodilatation, and impaired normal RPE function, and of which CSC is the most prominent disease. In patients with CSC, it has been found that pachychoroid may be caused by focal or diffuse dilated large choroidal vessels [22, 23], which is similar to our findings in the mouse choroid. CSC tends to have an acute onset, with spontaneous healing achieved in approximately 60% of patients [24]. This is consistent with the present observations, where damage to the RPE was repaired, to some extent, after 3 weeks. Although no epidemiological studies have explored the association between air pollution and CSC, a smoking habit is an independent risk factor for CSC [24], and previous studies have also found that cigarette smoking is related to increased subfovea choroidal thickness in patients with CSC [22, 25]. Since PM_2.5_, like the main components of cigarette smoke, can induce oxidative stress damage [31, 32], it can be hypothesized that acute PM_2.5_ exposure may be correlated with the development of CSC in conjunction with the findings in this study. In the future, well-designed cohort studies might be conducted to validate this association.

Unlike CSC, the association between PM_2.5_ exposure and AMD has been confirmed by numerous epidemiological studies [10–13]. Analysis of data from UK Biobank found higher odds of self-reported AMD among those exposed to higher PM_2.5_ levels, and that elevated PM_2.5_ concentrations were associated with a thinner RPE layer [11]. A Korean cohort study of older adults showed that nearly 5 years of air-pollution exposure was significantly related to increased morbidity of early AMD [12]. In addition, a decade-long cohort study in Taiwan revealed that long-term exposure to PM_2.5_ increased the risk of AMD development [13]. However, there were no previous studies that investigated the signs of AMD in animal models under PM_2.5_ exposure. In this study, the characteristic early pathological changes of AMD, including retinal neovascularization and Bruch's membrane thickening, were found in the retinas of mice exposed to PM_2.5_ for the first time (Fig. 5F), which strongly supports the view that long-term PM_2.5_ exposure is an important risk factor for the development of AMD. RPE is the main lesion site of AMD, and oxidative stress damage in RPE cells is the main mechanism causing RPE atrophy and loss of function [9, 33]. It is potent oxidants such as benzodiazepines and nicotine in cigarette smoke that make smoking a modifiable risk factor for AMD [34, 35]. Moreover, the exposure of mice to cigarette smoke has become a highly recognized way to establish AMD disease models, and more prolonged smoking exposure can induce more severe RPE apoptosis and retinal degeneration [36, 37]. Notably, the present study found that characteristic AMD changes in PM_2.5_-exposed mice were similar to those found in cigarette-smoke-exposed mice. Considering the similarity between PM_2.5_ and smoking-induced cellular damage [38], perhaps the oxidative damage in RPE cells is also an important pathological mechanism of PM_2.5_ exposure-induced AMD. Furthermore, it can be speculated that long-term exposure to PM_2.5_ has the potential to be used as a way to construct AMD animal models.

Several previous studies have been conducted to explore the pathological mechanisms of AMD triggered by PM_2.5_ exposure in cellular models [39–42]. These studies exposed ARPE-19 cells to appropriate concentrations of PM_2.5_ suspensions, thereby analyzing the mechanism of PM_2.5_ damage to RPE. The results showed that the cell viability of ARPE-19 cells decreased and epithelial-mesenchymal transition through the PI3K/AKT/mTOR pathway was promoted after PM_2.5_ stimulation [41]. It was also found that PM_2.5_ inhibited ciliogenesis in ARPE-19 cells, and primary ciliogenesis inducer effectively attenuated reactive oxygen species damage and inflammation [39, 40]. However, the major problem with these studies is that the RPE cellular model with direct PM_2.5_ exposure is not sufficiently realistic to reflect the in vivo microenvironment, mainly because the various layers of the ocular barrier prevent airborne as well as circulating PM_2.5_ from coming into direct contact with the RPE [43, 44]. In order to establish a more credible cellular model, the present study focused on the key pathological features of choroidal thickening and vasodilatation under PM_2.5_ exposure, and creatively simulated the choroidal vasodilatation process with cyclic mechanical stretching of HUVECs. We also investigated the alteration of the RPE by co-culturing stretching HUVECs with ARPE-19 cells. Recent studies have demonstrated that the mouse and monkey choroidal congestion model can be a reliable animal model of pachychoroid [45, 46], but to our knowledge there is no cellular model for this disease spectrum, and perhaps the introduction of a mechanical stretching device can provide a breakthrough to further investigate the molecular mechanism of oBRB injury under choroidal vasodilatation.

The reason for applying a co-culture cell model to study changes in the oBRB in response to PM_2.5_ exposure is that the choroid and RPE constitute a tightly linked complex [17, 27]. Together, they form the bulk of the oBRB and control the transportation of biological molecules between the retina and the blood. The RPE and choroid are highly interdependent during embryonic development. Previous experiments have revealed that the development of choriocapillaris depends on proper RPE differentiation [47], and choroidal vascular endothelial cells in turn secrete factors to remodel the TJ, basement membrane, and barrier function of the RPE [26]. The interaction between the RPE and choroid is not only limited to development, but is also significant in the maintenance of physiological states in adults. Histopathological studies found that choroidal dysfunction precedes RPE damage in wet AMD, whereas the causal relationship is reversed in dry AMD [48]. Various direct or indirect co-culture models based on primary, immortalized, and pluripotent stem cell-derived cells have been used to study the interaction between the RPE and choroid [49–54]. These studies indicated that direct contact between RPE cells and choroidal vascular endothelial cells promoted choroidal neovascularization [55, 56]. In turn, choroidal neovascularization drives RPE cells to proliferate and surround the new vessels in an attempt to reconstruct the oBRB [57, 58]. The present study is the first to introduce stretched vascular endothelial cells into an oBRB co-culture system, exploring the effects of choroidal hemodynamic changes on the RPE and supporting corroboration with findings on animal models under acute and continuous PM_2.5_ exposure.

There are some shortcomings in this study as well. First of all, our animal model does not fully reflect the reality although the calculation of exposure doses is based on actuality. In addition to the ocular surface, PM_2.5_ can enter the blood circulation through the respiratory tract to affect the eyes, so aerogel exposure would be a more authoritative model for animal experiments. Secondly, the description of the effects of PM_2.5_ exposure on the oBRB remained at the phenotypic level, without exploring the signaling pathways and molecular mechanisms involved. For example, oxidative damage and non-programmed cell death in RPE cells, and inflammatory and biomechanical pathways in choroidal vascular endothelial cells, are likely to be the essential pathological mechanisms. Identifying the key molecular targets will provide potential intervention guidance for air-pollution-related fundus diseases. Additionally, although the present study systematically described the respective changes of RPE and choroid in mice exposed to PM_2.5_, it is still not clear which of the two is the driving factor. The present cellular experiment investigated the effect of the choroid on the RPE, but no suitable cellular model has been established to simulate the effect of the RPE on the choroid, which deserves investigation in future studies.

## Conclusion

This study is the first to identify and describe the adverse effects of air pollution exposure on the oBRB. Choroidal vasodilatation and damage to the barrier function of the RPE in mice occurred after acute PM_2.5_ exposure, with an increasing risk of retinal edema. After continuous exposure, the damage to the RPE was repaired to some extent, but a series of early retinal degenerative changes was observed. These findings further elucidate the association of air pollution with retinal diseases such as CSC and AMD, and provide valuable insights into the benefits of early intervention.

## Materials and methods

### PM_2.5_ preparation and animal model

The method used to produce the PM_2.5_ samples has been elucidated in several other papers [14, 59–61]. In brief, the sample location was established in the yard of the Center for Disease Control and Prevention of Zhejiang Province in Hangzhou, Zhejiang, China, at a height equivalent to the respiratory zone. Samples of atmospheric PM_2.5_ were taken using 90-mm quartz fiber filters. Following collection, the quartz filters were sonicated for 30 min while being immersed in 75% ethanol. The suspensions obtained were lyophilized and dried to a final concentration of 2 mg/mL before being re-suspended in sterile water. Aliquots of the PM_2.5_ suspensions were then kept at − 80 °C for use in upcoming experiments.

All animal study was approved by the Second Affiliated Hospital of Zhejiang University School of Medicine’s Animal Ethics Committee. Male C57BL/6J mice that were 8 weeks old were chosen for the experiments (Slac Laboratory Animal Co. Ltd, Shanghai, China). The mice were housed in a typical setting and divided into 3 groups at random. As shown in Fig. 1A, during the exposure period, both eyes of mice in the PM_2.5_ exposure group received manual applications of 3 μL PM_2.5_ in PBS (1 mg/mL) 4 times daily. The PM_2.5_ level matched various other animal studies and was consistent with our earlier studies [14, 59, 62–64]. The precise approach to calculating PM_2.5_ concentration was presented in Additional file 2: Text S1. 3 μL PBS was given to the control group 4 times per day and the blank group had no manual treatment applied.

### Optical coherence tomography angiography

OCT is a non-invasive tomography technique that uses the light interference combined with computerized imaging technology to display tomographic images of fundus tissues. OCTA, which is based on OCT technology, can display retinal and choroidal blood flow patterns and changes. Detailed instrument parameters and operating methods have been described in a previous study [14, 65–67]. In brief, the mice were immobilized in a 3D-printed holder under anesthesia with dilated pupils, then tomographic and blood flow images were acquired before and after PM_2.5_ exposure and quantified by an algorithm to compare parameters such as vessel diameter and density.

### Retinal hydration rate assay

After the mice were sacrificed, the eyeballs were enucleated and cut along the scleral margin. After the lenses were removed, the retinas were dissociated from the eyeball wall and detached from the optic nerve with tweezers, and the whole retinas were removed and placed in microcentrifuge tubes. The tubes, with caps open, were heated at 60 °C for 6 h in a metal bath. In this process, the weight of the empty tube (t) and the weight of tube with the retina before (b) and after heating (a) were measured by microbalance. The hydration rate of the retina can be calculated by substituting the formula (b − a)/(b − t).

### HE staining

The mice’s eyeballs were excised and fixed in 4% paraformaldehyde at 4 °C for 15 h after being sacrificed. After the eyes were dehydrated and paraffin-embedded, serial sections were cut every 5 microns in the sagittal plane, where the optic nerve resides. Each section was spaced 30 μm apart and stained with hematoxylin and eosin. Images were acquired using an optical microscope (Olympus, Tokyo, Japan). ImageJ software (National Institutes of Health, MD, USA) was applied to measure the thickness of the choroid and RPE layers.

### Transmission electron microscope

Mouse retinas were fixed with 1% OsO4 after being fixed in 2.5% glutaraldehyde in a 0.1 M phosphate buffer for over 6 h. Next, they were dehydrated with graded alcohol, embedded in acetone with 33% epoxy resin, acetone with 67% epoxy resin, and epoxy resin. A 120 kV transmission electron microscope (TEM; Tecnai G2 Spirit, Massachusetts, USA) was used to study slimline slices that were mounted on copper grids and stained with uranyl acetate and lead citrate. ImageJ software was used to determine the thickness of the Bruch’s membrane.

### Co-culture cell model

To simulate the effect of dilated choroidal vessels on the RPE during PM_2.5_ exposure, stretched HUVECs and ARPE-19 cells were employed to construct an indirect co-culture model (Fig. 6A). The entire culture phase was completed in a 37 °C incubator with 5% CO_2_. First, HUVECs were seeded onto stretch chambers (2 cm × 2 cm; Menicon Life Science, Nagoya, Japan) with high cell adhesion properties and cultured in DMEM medium (Gibco, CA, USA) with 10% fetal bovine serum (Gibco, CA, USA). After the HUVECs had grown and completely covered the bottom, the chamber was stretched cyclically using a ShellPa Pro Cell Stretching System (Menicon Life Science, Nagoya, Japan) and the adherent HUVECs on the chambers underwent a fixed frequency of stretching. In order to accurately simulate the in vivo changes of choriocapillaris endothelial cells, the stretching period was set to 1 s, the amplitude was 10% of the original length, and the total duration was 12 h. After stretching, the HUVECs media was collected, cleared by centrifugation (400 g, 3 min) and kept at − 80 °C as conditioned media for further experiments.

ARPE-19 cells were seeded onto dishes and cultured in DMEM/F12 medium (Gibco, CA, USA) with 20% fetal bovine serum (Gibco, CA, USA). After ARPE-19 cells adhered and covered 70% of the bottom area of the dishes, half of the original culture medium was replaced with conditioned media from the stretched HUVECs. Then, co-culturing of 24 h and 48 h, respectively, was undertaken to simulate acute and continuous PM_2.5_ exposure. The conditioned medium in the control group was derived from unstretched HUVECs in parallel with the experimental group, while the ARPE-19 cell-culturing process in the blank group did not involve conditioned medium.

### Paracellular permeability assays

Permeability assays were conducted with ARPE-19 cells cultured in Transwell inserts and co-cultured with conditioned media (Fig. 6C). After being washed with PBS, the insert’s top and bottom chambers received 0.5 mL of 1 M Lucifer Yellow lithium salt (457.2 Da; Molecular Probes, CA, USA) and 1 mL PBS, respectively. After 2 h of incubation at 37 °C, 100 mL aliquots from the bottom chamber were transferred to a 96-well plate, and the absorbance was assessed using an iMark Absorbance Reader (Bio-Rad, CA, USA) at 450 nm.

### Cell biological properties assays

The viability, proliferation, and migration of ARPE-19 cells co-cultured with conditioned media were tested. To detect viability, 10 µL Cell Counting Kit-8 (Dojindo, Kumamoto, Japan) solution was added in 100 µL media per well. After 2 h of incubation at 37 °C, absorbance at 450 nm of the 96-well plate was detected by an iMark Absorbance Reader (Bio-Rad, CA, USA).

The BrdU assay was used to detect cell proliferation capacity. 1 mg/ml of BrdU (5-iodine-2'-deoxyuridine; Sigma Aldrich, MO, USA) was added to a 24-well plate incubated with ARPE-19 cells for 6 h. Subsequently, cells seeded on cover glasses were fixed in 4% paraformaldehyde for 20 min and then treated with 2 mol/L hydrochloric acid (HCL) for 10 min. The cells were permeabilized with PBS containing 0.4% Triton X-100 (Sigma-Aldrich, MO, USA) for 20 min and incubated with a BrdU primary antibody overnight at 4 °C before incubation with a secondary antibody at room temperature and protected from light for 1 h. Finally, nuclei were stained with 1 μg/mL 4,6-diamido-2-phenylindole dihydrochloride (DAPI; Sigma-Aldrich, MO, USA). The percentage of BrdU-positive cells was calculated after image acquisition with a Leica TCS SP8 confocal microscope (Leica, Wetzla, Germany).

Scratch wound-healing assay was conducted to evaluate cell migration. A 200 µL pipette was used to draw a uniform straight line across the monolayer after ARPE-19 cells had grown over the six-well plate into a monolayer. After rinsing off the suspended cells with PBS, cells were grown in serum-free conditioned medium. The ratio of wound area to initial wound area at different time points was recorded and calculated under an Olympus light microscope (Olympus, Tokyo, Japan).

### Immunofluorescence staining

The immunofluorescence staining of the retina and RPE flat-mounts of mice were prepared as follows. The corneas and lenses of the eyes fixed in 4% paraformaldehyde for 24 h were removed first, and then the neural retinas and the remaining posterior segments were separated from each other. The 2 parts were both cut from the edge centripetally by 2/3 of the radius, and they were cut into a quatrefoil shape and laid flat for subsequent staining. The upper surface of the remaining posterior segment was the RPE layer. The posterior segments were first soaked with PBS containing 0.5% Triton X-100 (Sigma-Aldrich, MO, USA) for 20 min for permeabilization, then soaked with blocking solution (5% goat serum, 2% BSA, and 0.1% Tween-20 in PBS) for 1 h, followed by overnight incubation with a primary antibody (Additional file 2: Table S1) at 4 °C; they were then incubated with a fluorescent secondary antibody (Additional file 2: Table S1) for 1 h. Finally, the nuclei were stained with 1 μg/mL DAPI for 10 min, and then covered with coverslips to be photographed under a Leica TCS SP8 confocal microscope (Leica, Wetzla, Germany).

For staining of the co-cultured ARPE-19 cells, the cells were seeded on autoclaved cover glass. Following co-culturing, ARPE-19 cells were washed with PBS and fixed with 4% paraformaldehyde. The remaining steps were essentially the same as in the fluorescent staining method for the RPE flat-mounts, detailed above.

### Western blotting

In the mouse eyes, neural retinas and anterior segments were removed, and the RPE/choroid tissues were mechanically separated from the sclera using a microblade. 4 eyes’ worth of tissue were collected as 1 sample and carefully dissected by ultrasound. After being extracted using a Protein Extraction Kit (Sangon, Shanghai, China), protein samples from mouse RPE/choroid tissues or ARPE-19 cells were collected and quantified using a Pierce BCA Protein Assay Kit (Thermo Fisher Scientific, MA, USA). On 10% SDS PAGE Gels (YaMei, Shanghai, China), identical quantities of protein were electrophoresed before being transferred to PVDF membranes (Millipore, NJ, USA). Membranes were then blocked with Protein Free Rapid Blocking Buffer (YaMei, Shanghai, China). After being blocked, the PVDF membranes were immersed with primary antibodies (Additional file 2: Table S1) at 4 °C overnight and incubated in secondary antibodies (Additional file 2: Table S1) for 1 h at room temperature. Glyceraldehyde-3-phosphate dehydrogenase (GAPDH) served as a reference and the relative expressions of the proteins were detected and assessed by measuring the grayscale value of the bands using Image J software (National Institutes of Health, DC, USA). All blot images used in the article were presented in Additional file 1.

### Statistical analysis

Unless otherwise stated, all data were presented as mean standard deviation (SD), with *p* < 0.05 denoting statistical significance, and every experiment was conducted at least 3 times. The figure legends include the number of times each experiment was done. GraphPad Prism 8.0 (GraphPad Software, CA, USA) was used to analyze comparisons between several groups using one-way ANOVA.

### Supplementary Information

**Additional file 1:** All blot images used in the article.

**Additional file 2:** Antibodies list and calculation method of PM_2.5_ concentration for animal experiments.

## Data Availability

All data generated or analyzed during this study are included in this published article [and its Additional files].
