# Supplementary material for: Acute and continuous exposure of airborne fine particulate matter (PM2.5): diverse outer blood–retinal barrier damages and disease susceptibilities
Source: Part Fibre Toxicol. 2023 Dec 18;20:50. doi: 10.1186/s12989-023-00558-2 (PMC10726629; doi:10.1186/s12989-023-00558-2)
Supplement: Supplementary file 1 — Additional file 1: All blot images used in the article. [file 12989_2023_558_MOESM1_ESM.pptx]

## Slide 1
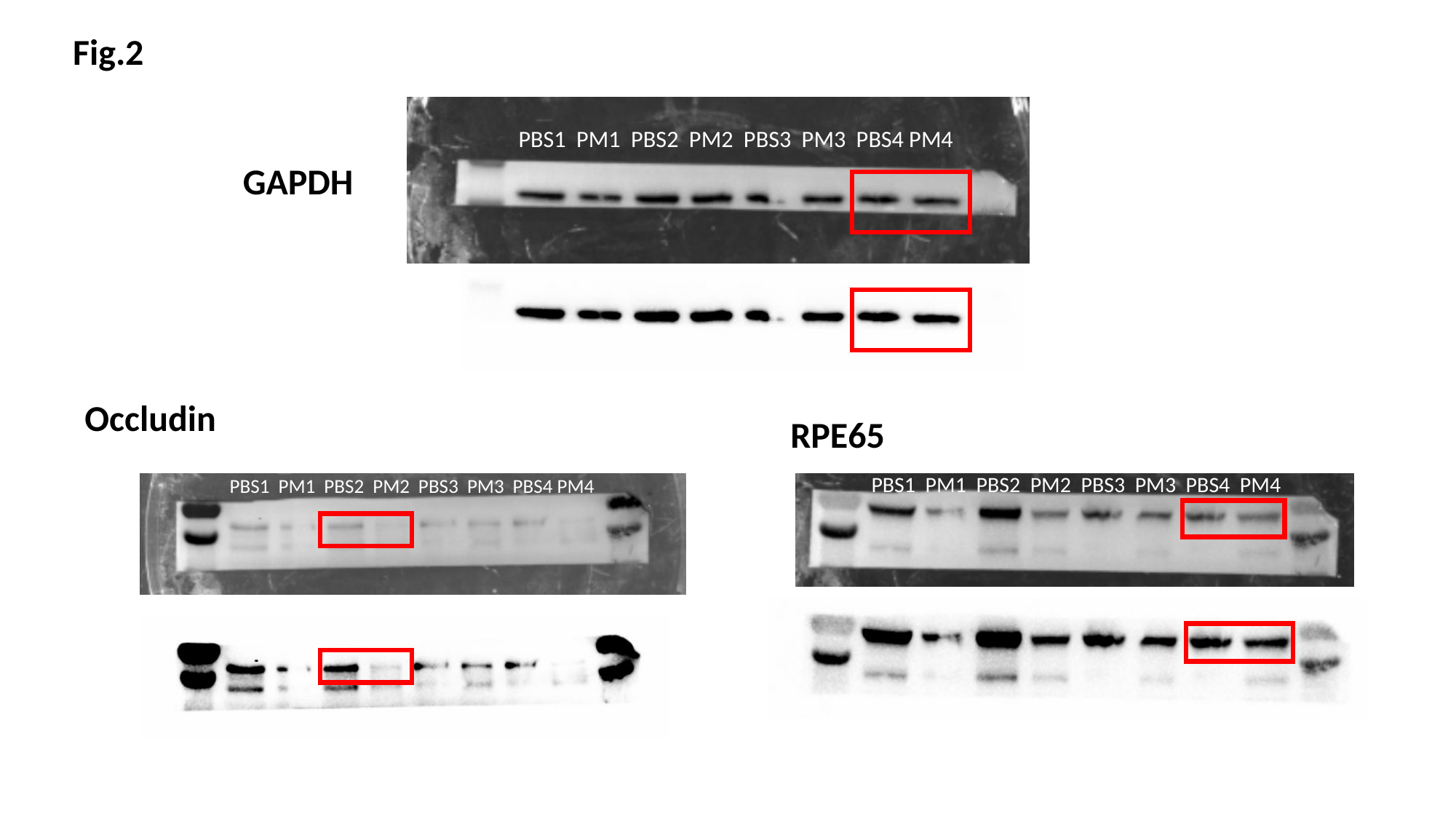

Fig.2
PBS1 PM1 PBS2 PM2 PBS3 PM3 PBS4 PM4
GAPDH
Occludin
RPE65
PBS1 PM1 PBS2 PM2 PBS3 PM3 PBS4 PM4
PBS1 PM1 PBS2 PM2 PBS3 PM3 PBS4 PM4

## Slide 2
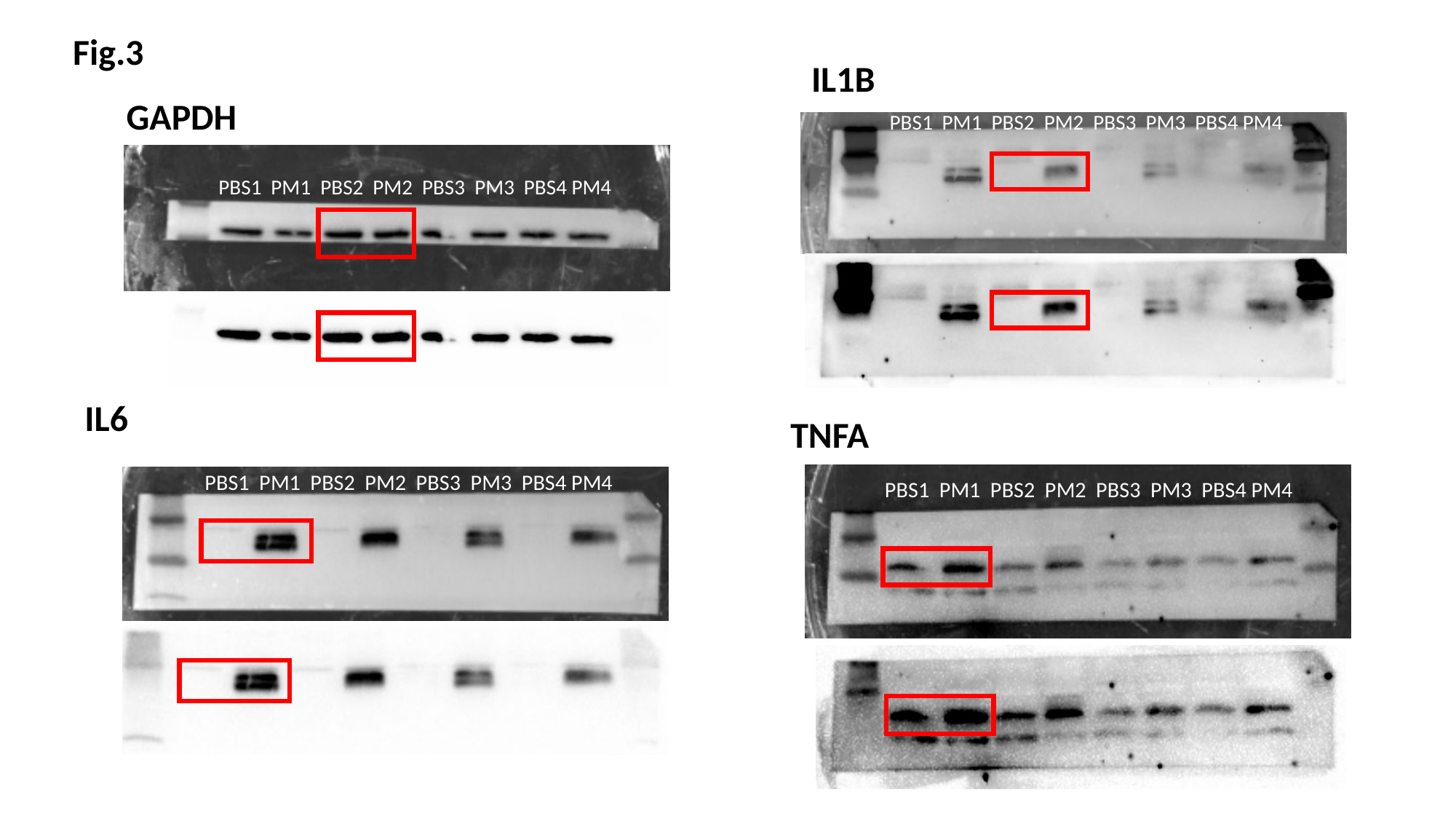

Fig.3
IL1B
GAPDH
PBS1 PM1 PBS2 PM2 PBS3 PM3 PBS4 PM4
PBS1 PM1 PBS2 PM2 PBS3 PM3 PBS4 PM4
IL6
TNFA
PBS1 PM1 PBS2 PM2 PBS3 PM3 PBS4 PM4
PBS1 PM1 PBS2 PM2 PBS3 PM3 PBS4 PM4

## Slide 3
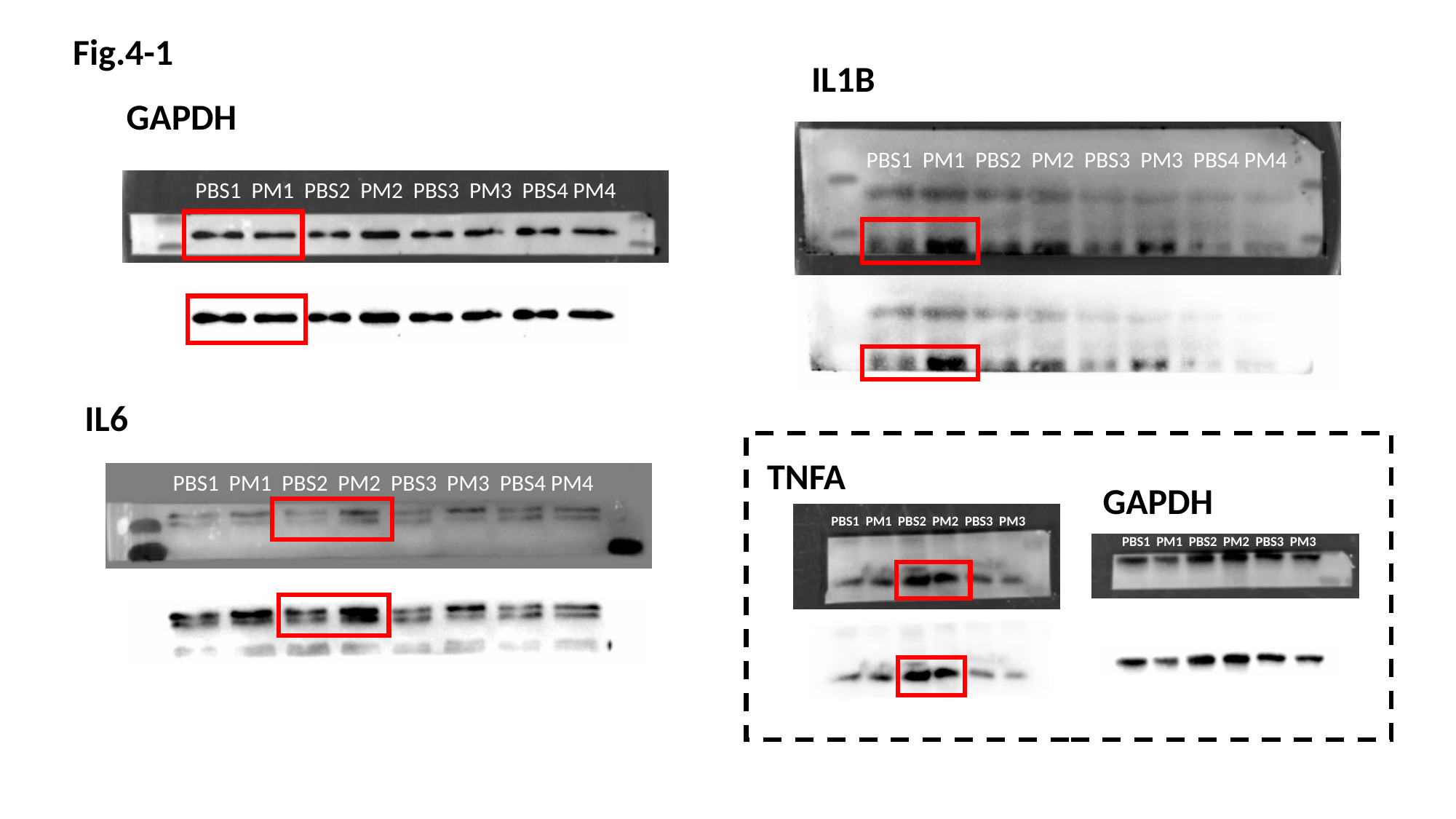

Fig.4-1
IL1B
GAPDH
PBS1 PM1 PBS2 PM2 PBS3 PM3 PBS4 PM4
PBS1 PM1 PBS2 PM2 PBS3 PM3 PBS4 PM4
IL6
TNFA
PBS1 PM1 PBS2 PM2 PBS3 PM3 PBS4 PM4
GAPDH
PBS1 PM1 PBS2 PM2 PBS3 PM3
PBS1 PM1 PBS2 PM2 PBS3 PM3

## Slide 4
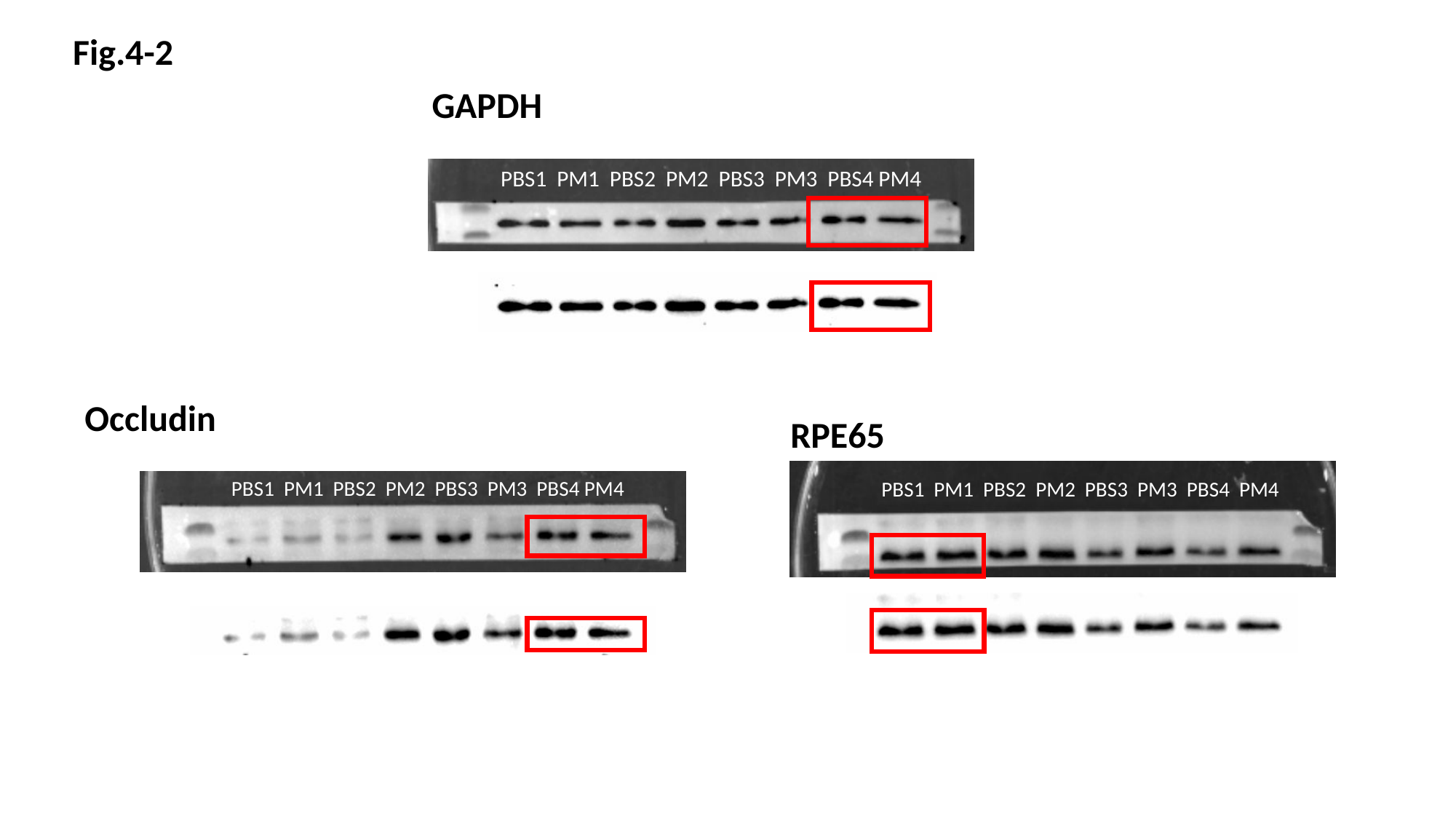

Fig.4-2
GAPDH
PBS1 PM1 PBS2 PM2 PBS3 PM3 PBS4 PM4
Occludin
RPE65
PBS1 PM1 PBS2 PM2 PBS3 PM3 PBS4 PM4
PBS1 PM1 PBS2 PM2 PBS3 PM3 PBS4 PM4

## Slide 5
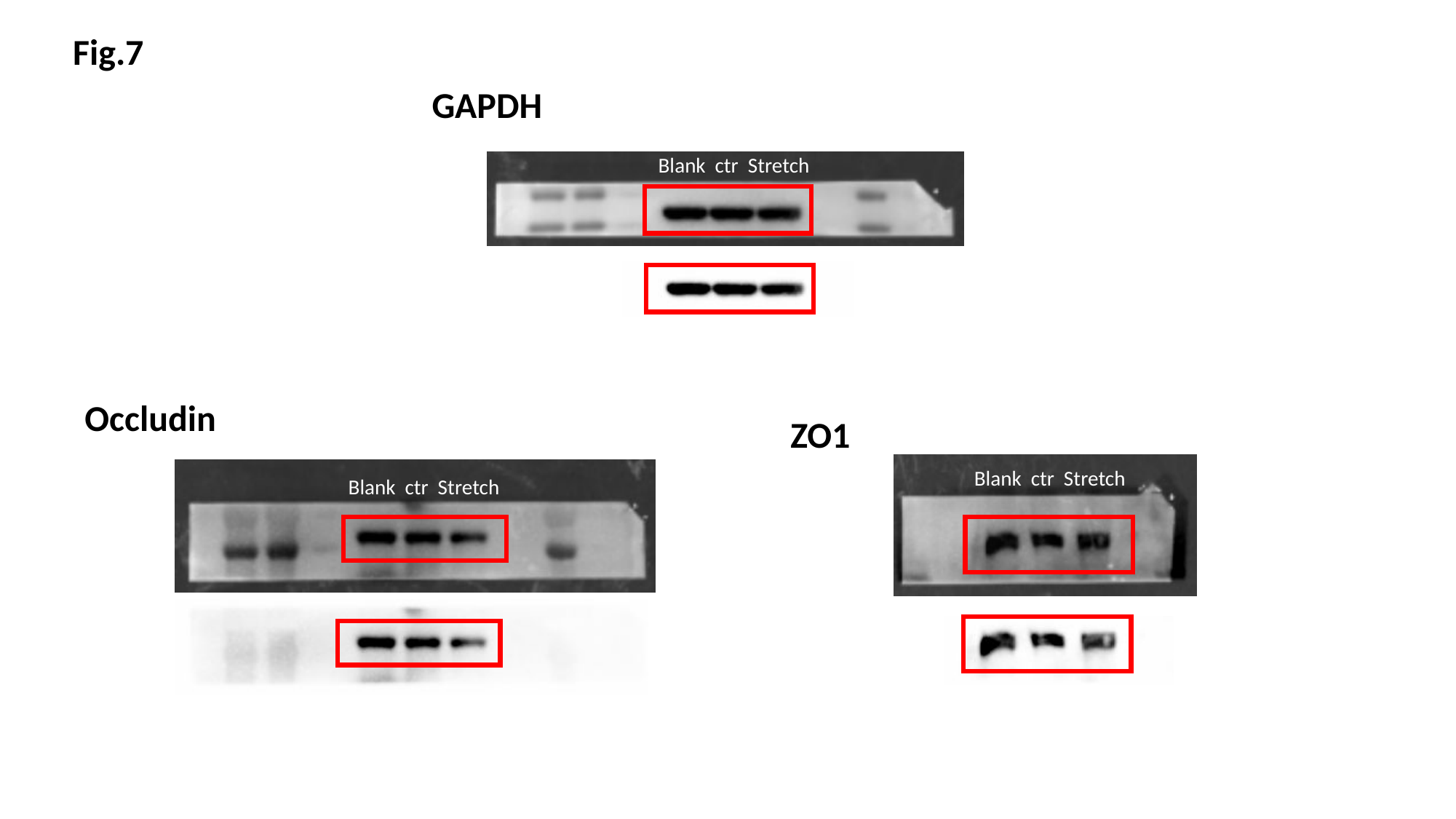

Fig.7
GAPDH
Blank ctr Stretch
Occludin
ZO1
Blank ctr Stretch
Blank ctr Stretch

## Slide 6
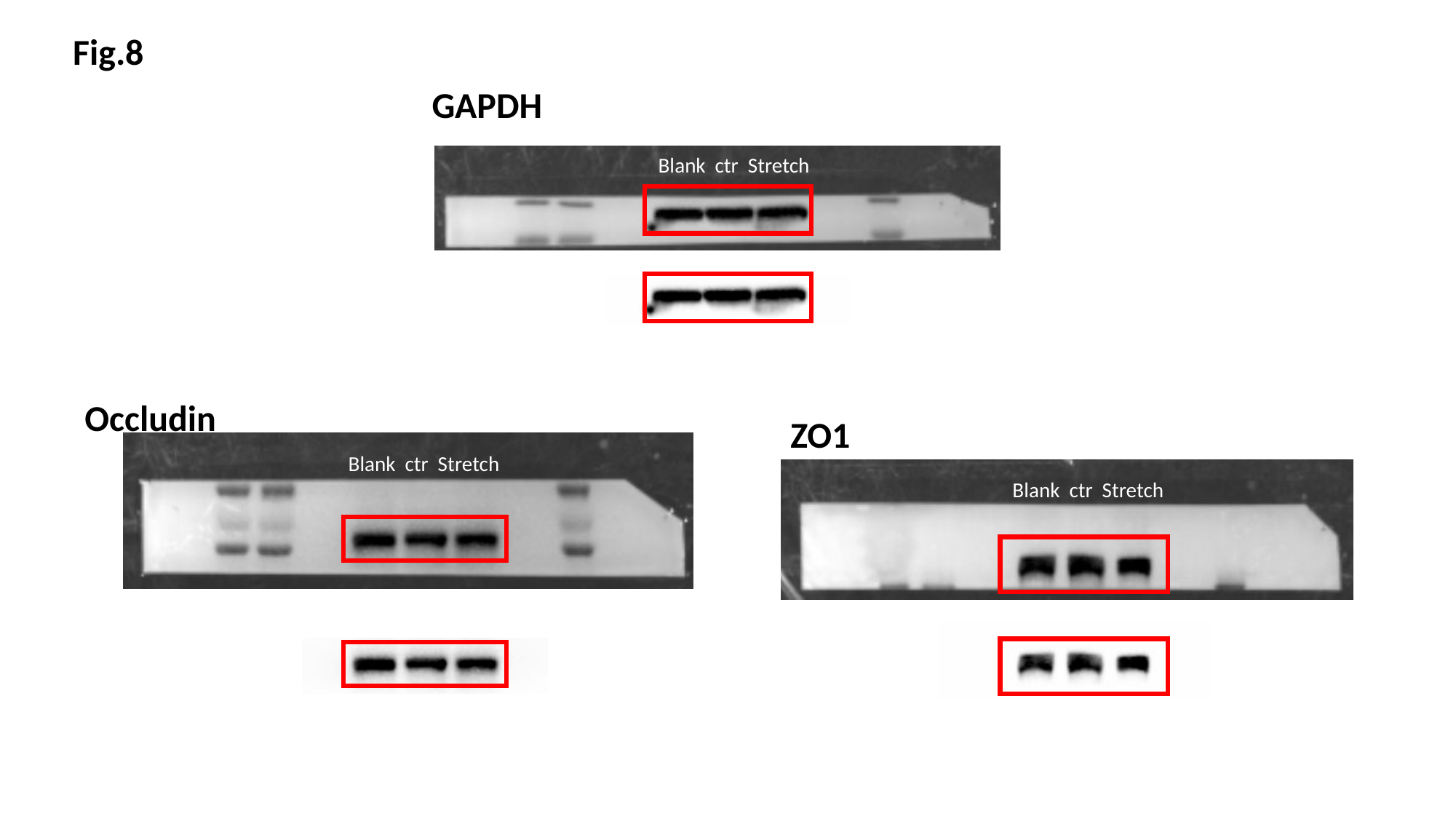

Fig.8
GAPDH
Blank ctr Stretch
Occludin
ZO1
Blank ctr Stretch
Blank ctr Stretch
