# Supplementary material for: Acute and continuous exposure of airborne fine particulate matter (PM2.5): diverse outer blood–retinal barrier damages and disease susceptibilities
Source: Part Fibre Toxicol. 2023 Dec 18;20:50. doi: 10.1186/s12989-023-00558-2 (PMC10726629; doi:10.1186/s12989-023-00558-2)
Supplement: Supplementary file 2 — Additional file 2: Antibodies list and calculation method of PM2.5 concentration for animal experiments. [file 12989_2023_558_MOESM2_ESM.docx]

**Supplementary Materials**

**Acute and continuous exposure of airborne fine particulate matter (PM_2.5_): Diverse outer blood–retinal barrier damages and disease susceptibilities**

Yuzhou Gu^#^, Feiyin Sheng^#^, Mengqin Gao^#^, Li Zhang, Shengjie Hao, Shuying Chen, Rongrong Chen, Yili Xu, Di Wu, Yu Han, Lu Chen, Ye Liu, Bing Lu, Wei Zhao, Xiaoming Lou, Zhijian Chen, Peng Li*, Xiaofeng Wang*, Ke Yao*, Qiuli Fu*

**Content**

**Table S1 Antibodies for and immunofluorescence staining and Western blotting**

**Text S1 Calculation method of PM_2.5_ concentration for animal experiments**

**Table S1 Antibodies for and immunofluorescence staining and Western blotting**

| Antibody | Company | Category number | Dilution |
| --- | --- | --- | --- |
| Primary antibody |  |  |  |
| Rabbit anti-ZO1 | Abcam | ab96587 | 1:200 (IF)  1:1000 (WB) |
| Rabbit anti-Occludin | Abcam | ab216327 | 1:100 (IF)  1:1000 (WB) |
| Rabbit anti-RPE65 | Abcam | ab231782 | 1:250 (IF)  1:1000 (WB) |
| Mouse anti-IL1B | CST | 12242 | 1:1000 (WB) |
| Rabbit anti-IL6 | CST | 12912 | 1:1000 (WB) |
| Rabbit anti-TNF-α | CST | 11948 | 1:1000 (WB) |
| Rabbit anti-BrdU | Abcam | ab152095 | 1:50 |
| Rabbit anti-GAPDH | CST | 5174 | 1:5000 (WB) |
| Secondary antibody |  |  |  |
| HRP-conjugated anti-rabbit IgG | CST | 7074 | 1:5000 (WB) |
| HRP-conjugated anti-mouse IgG | CST | 7076 | 1:5000 (WB) |
| Anti-rabbit IgG(Alexa Fluor 555 Conjugate) | CST | 4413 | 1:1000 (IF) |

**Text S1 Calculation method of PM_2.5_ concentration for animal experiments**

The rationale for the exposure treatment in cell and rat models is explained below. According to the data provided by the National Ministry of Environmental Protection of China and Environmental Protection Agency of US, 100~250 μg/m^3^ was selected as the PM concentration of mild PM_2.5_ pollution. Taking an adult engaged in indoor work as an example, the average radius of an adult’s cornea is approximately 6 mm. The estimate duration of daily outdoor activity is set as 2 hours (7200 seconds) and the movement velocity of an adult with light activity is estimated as 1 m/s. Then the mass of daily PM_2.5_ exposure in is calculated as follows:

mass of daily PM_2.5_ exposure = V ∙ c = S ∙ v ∙ t ∙ c

= π × (0.006 m)^2^ × 1m/s × 7200 s × (100~250) μg/m^3^

≈ (80~200) μg

mass - mass of PM_2.5_ contact with one cornea per hour

V - volume of air contact with one cornea per hour

c - concentration of airborne PM_2.5_

S - surface area of the cornea

v - movement velocity of a person in light activity

t - PM_2.5_ exposure time

m - meter

s - second

m^3^ - cubic meter

The equal exposure mass in a mouse should be 5~12.5 μg, considering that the average radius of a mouse’s cornea is approximately 1.5 mm. In this study, the daily PM_2.5_ exposure mass in a rat is calculated as followed:

1 mg/mL × 3 μL ×4 times/day = 12 μg
